# Effect of Grinding Process Parameters and Storage Time on Extraction of Antioxidants from Ginger and Nutmeg

**DOI:** 10.3390/molecules27217395

**Published:** 2022-10-31

**Authors:** Szymon Poliński, Patrycja Topka, Małgorzata Tańska, Sylwia Kowalska, Sylwester Czaplicki, Aleksandra Szydłowska-Czerniak

**Affiliations:** 1 Department of Analytical Chemistry and Applied Spectroscopy, Faculty of Chemistry, Nicolaus Copernicus University in Toruń, 87-100 Toruń, Poland; 2 Confectionery Factory “Kopernik” S.A., 87-100 Toruń, Poland; 3 Department of Food Plant Chemistry and Processing, Faculty of Food Sciences, University of Warmia and Mazury in Olsztyn, 10-718 Olsztyn, Poland

**Keywords:** vegetal spices, grinding process, antioxidant capacity, phenolic acids, Box–Behnken design, response surface methodology

## Abstract

The aim of this study was to optimize the grinding process parameters (mesh size of grinder sieve (X_1_), the peripheral velocity of the grinding wheels (X_2_)), and the storage time (X_3_) of ground ginger rhizome and nutmeg to obtain ethanol and ethanol-water extracts with improved antioxidant properties. The optimal conditions were estimated using response surface methodology (RSM) based on a three-variable Box–Behnken design (BBD) in order to maximize the antioxidant capacity (AC) determined by the 2,2-diphenyl-1-picrylhydrazyl (DPPH) and 2,2’-azino-bis(3-ethylbenzothiazoline-6-sulfonic acid) (ABTS) methods, and the total phenolic content (TPC) was determined by the Folin–Ciocalteu (F–C) method in spice extracts. Additionally, the phenolic acid profiles in extracts from optimized conditions were analyzed using ultra-performance liquid chromatography (UPLC). It was found that the optimal preparation conditions for antioxidant extraction were dependent on the spice source and solvent type. The best antioxidant properties in nutmeg extracts were achieved for X_1_ = 1.0 mm, X_2_ = 40–41 Hz and X_3_ = 7 days, whereas the optimized parameters for ginger extracts were more varied (1.0–2.0 mm, 43–50 Hz and 1–9 days, respectively). The ginger extracts contained 1.5–1.8 times more phenolic acids, and vanillic, ferulic, gallic, and *p*-OH-benzoic acids were dominant. In contrast, the nutmeg extracts were rich in protocatechuic, vanillic, and ferulic acids.

## 1. Introduction

The first evidence of using spices for culinary purposes comes from the Neolithic era [1]. In ancient times, spices were used for culinary rituals, perfumery, and medicine. During the Middle Ages, the culinary role of spices increased significantly, retaining their importance in medicinal applications [2]. Geographical discoveries and trade development contributed to increased spice popularity beginning in the 16th century, and the number of uses for spices in food, medicine, and perfumery multiplied during this time period [3]. Nowadays, spices are an inherent part of all known cuisines, but it is obvious that spices are far more important in some cuisines than others [4]. “Spices” is a culinary term, not one of the botanical categories, and does not refer to a specific part of a plant or plant species [5]. Spices can be sourced from different parts of plants, such as leaves, buds, bark, roots, berries, seeds, and flowers.

One of the best-known spices can be counted as ginger (*Zingiber officinale* Roscoe) rhizome, which is a perennial plant native to Southern Asia. Spice produced from ginger rhizome is widely used as a spice due to its characteristic pungency and piquant flavor. However, ginger rhizome also plays a considerable role in medicine. This well-known spice has a long history of use in Chinese and Ayurvedic medicine as an antiemetic, antipyretic, and anti-inflammatory agent [6]. Nowadays, there are many proven medical properties of ginger rhizome, such as anticarcinogenic, immune-modulatory, antibacterial, antifungal, anti-hyperglycaemic, antiviral, antipyretic, analgesic, and antiatherosclerotic activity. Furthermore, ginger is known to increase the motility of the gastrointestinal tract [7,8,9,10]. Ginger rhizome also indicated high antioxidant properties and considerable amounts of polyphenols, which are positively correlated [11,12,13].

Another widely used and highly interesting spice from a medical point of view is nutmeg. Nutmeg is a spice obtained from the dried seeds of the nutmeg tree (*Myristica fragrans*), which originally came from the Maluku Province of Indonesia [14]. It is often used as a spice in cuisines because of its sweet, spicy, and nutty taste, but due to its healing properties, it has also found application in natural medicine. Nutmeg has a variety of important health benefits, including brain stimulation, heart function stimulation, detoxification properties, insomnia treatment, toothache treatment, and anti-inflammatory properties [15]. There is also a scientific rationale for the traditional use of nutmeg in the management of male sexual disorders [16]. However, the consumption of nutmeg in certain doses causes toxic and narcotic effects [17,18,19]. Moreover, the prolonged abuse of nutmeg can lead to chronic psychosis, which can be identified by impaired thinking and emotions [20]. The toxic properties of nutmeg follow from the myristicin contained in this spice. Given that no holistic treatment for nutmeg intoxication has been developed, higher doses of nutmeg should be avoided [21]. As in the case of ginger, nutmeg indicates antibacterial, antifungal, and antioxidant properties [22,23].

Most spices are usually added to savory dishes, but ginger rhizome and nutmeg also appear as ingredients in recipes for sweet pastries and desserts. One of the well-known applications of both spices is in gingerbread production. These spices need to be subjected to a grinding process before their use. It is known that grinding processes alter the physical, chemical, functional, structural, and biological properties of raw materials. Moreover, obtained powders with smaller particle sizes promote the release of bioactive compounds and increase their antioxidant activity [24]. Archana et al. [25] reported that the grinding changed the crystal structure, internal cohesion, and interplanar distance of the nanocrystalline ginger powder. Moreover, the phenolic amounts, antioxidant properties, and superoxide radical scavenging of ginger powder increased due to changes in the crystalline structure, surface morphology, and large surface-to-volume ratio. Furthermore, volatile oil and oleoresin content (non-volatile resinous fraction comprising heat components, fixative, natural antioxidants, and pigments) were substantially different for nutmeg ground by ambient, chilled, and liquid nitrogen methods. Moreover, grinding equipment and differences in the cracking degree of the original nutmeg samples influenced the particle size of ground spice. At the same time, the temperature did not affect the particle size and uniformity of the ground nutmeg [26]. Spice grinding is one of the key processes in gingerbread production. Nevertheless, due to the grinding process improving the physicochemical and functional characteristics of the prepared spice powders, they can be suitable in the food, pharmaceutical, and medicine industries, and other related sectors for the development of new formulas of functional foods rich in bioactive compounds and new composite or functional materials [24]. For instance, ginger powders obtained by biological and conventional agricultural practices were used to design emulsions by employing Pickering particles that act both as physical emulsion stabilizers and as interfacial reservoirs of bioactive compounds. The proposed ginger powder-based Pickering emulsions had high stability to oxidation and promising antioxidant and α-amylase inhibitory activity [27]. Moreover, the addition of ginger powder to wheat bread and cooked pork burgers markedly increased the antioxidant properties and functionality of these enriched products [28,29]. Consequently, ginger powder as an ingredient in the formulation of the burgers reduced lipid oxidation and the total saturated fatty acids in burgers, increasing the nutritional values of these ready-to-cook products [29].

Often, spices are converted to powders by the traditional mechanical process of grinding, which leads to an increase in temperature as high as 43–95 °C [30]. The study of Makanjuola [31] suggests that particle size influences the extraction of antioxidants. Furthermore, the optimum powder size that would maximize antioxidant extraction may be dependent on the solvent used and the antioxidant property being measured.

Although the antioxidant activity and phenolic compounds contents of herbs and spices have been extensively studied, a study on the effect of time after grinding on antioxidant properties is still lacking. Taking into account the significance of the grinding process for ginger and nutmeg antioxidant properties, it is expected that optimization of this process can affect the health-promoting properties of these spices and thus gingerbreads that contain them. Therefore, this work is focused for the first time on the optimization of grinding process parameters and the storage time of ground ginger rhizome and nutmeg to obtain their ethanol and ethanol-water extracts with high antioxidant properties. Variables such as the mesh size of the grinder sieve (X_1_), the peripheral velocity of the grinding wheels (X_2_), and the storage time (X_3_) were optimized to enhance the antioxidant potential of the ground spices by using response surface methodology (RSM) with a Box–Behnken design (BBD). Moreover, modified spectrophotometric methods, 2,2-diphenyl-1-picrylhydrazyl (DPPH), 2,2’-azino-bis(3-ethylbenzothiazoline-6-sulfonic acid) (ABTS), and the Folin–Ciocalteu (F–C) test were used to determine the antioxidant capacity (AC) and total phenolic content (TPC) in ethanol and ethanol-water extracts of the prepared spice powders stored in air for different periods. However, ultra-performance liquid chromatography (UPLC) coupled with a triple quadrupole mass spectrometer was applied to profile the phenolic acids in ginger rhizome and nutmeg extracts obtained under optimal conditions.

## 2. Results and Discussion

### 2.1. Effect of Grinding Process and Storage Time on Antioxidant Properties of Ginger Rhizome and Nutmeg

The AC and TPC (experimental and predicted) results of ethanol and ethanol-water extracts of ground ginger rhizome and nutmeg are listed in Table 1 and Table 2, respectively.

Significant differences in the mean values of AC and TPC in the ethanol and ethanol-water extracts of ginger rhizome and nutmeg were observed (Tukey’s test, Table 1 and Table 2).

As can be seen in Table 1, the grinding process of ginger rhizome influenced the highest DPPH method result for ethanol-water extraction (DPPH = 189.9 mmol TE/100 g) when a 1.4 mm mesh size grinder sieve was used, the highest peripheral velocity of grinding wheels (50 Hz) was set, and the storage time was 14 days. However, the lowest results of the DPPH method for extraction with ethanol (DPPH = 22.5 mmol TE/100 g) were achieved for a 2.0 mm mesh size, 40 Hz peripheral velocity, and a 0-day storage time, while for extraction with ethanol-water (DPPH = 26.4 mmol TE/100 g) for a 2.0 mm mesh size, 30 Hz peripheral velocity, and a 7-day storage time.

Unexpectedly, the ethanol-water extraction of freshly ground ginger rhizome using 30 Hz peripheral velocity and a 1.4 mm mesh size caused the lowest ABTS method result (ABTS = 80.4 mmol TE/100 g). It is worth noting that the highest ABTS results for this spice were obtained using a 1.0 mm mesh size grinder, 30 Hz peripheral velocity, and a 7-day storage time (454.0 and 440.0 mmol TE/100 g for the ethanol and ethanol-water extracts, respectively). This suggests that the results of the antioxidant properties are related to the degree of spice sample granulation.

It can be noted that the highest TPC result analyzed by the F–C method (507.4 mg GA/100 g) was obtained for the ethanol extraction of phenolic compounds from ginger rhizome ground by a 1.0 mm mesh size grinder, using the highest peripheral velocity of grinding wheels (50 Hz) and stored for 7 days. Replacing the extraction solution with ethanol-water in the case of ginger rhizome ground in a grinder with a larger mesh (1.4 mm) and slower peripheral velocity of the grinding wheels (30 Hz) resulted in the lowest amount of polyphenols in the fresh sample (TPC = 81.0 mg GA/100 g).

On the other hand, the highest DPPH result (230.0 mmol TE/100 g) was obtained for the ethanol extract of nutmeg ground at the lowest peripheral velocity of grinding wheels (30 Hz), sieved by a 1.0 mm mesh size grinder sieve, and stored for 7 days. However, the lowest DPPH results revealed ethanol and ethanol-water extracts (29.8 and 28.3 mmol TE/100 g, respectively), prepared from fresh ground nutmeg using a 40 Hz peripheral velocity of the grinding wheels and sieved to the largest particle size (2.0 mm).

Compared with the DPPH results, only an increase in the peripheral velocity of the grinder to 50 Hz caused the highest ABTS values for ethanol (634.0 mmol TE/100 g) and ethanol-water (657.4 mmol TE/100 g) extracts of nutmeg. However, a decrease in the peripheral velocity of grinding wheels to 30 Hz and using the largest sieve mesh (2.0 mm) resulted in the lowest ABTS results (62.3 and 147.5 mmol TE/100 g) in both nutmeg extracts (Table 2).

Similarly, the lowest TPC results in the nutmeg ethanol and ethanol-water extracts (125.9 and 148.2 mg GA/100 g, respectively) were observed for samples freshly ground at a lower peripheral velocity of the grinding wheels (30–40 Hz) and sieved through larger mesh sizes (1.4–2.0 mm). Nevertheless, the highest TPC result (511.1 mg GA/100 g) in the ethanol extract of nutmeg was found at the central point of BBD, whereas the ethanol-water extract of fresh nutmeg ground using a medium peripheral velocity of grinder (40 Hz) and sieved by the lowest mesh size (1.0 mm) was the richest source of polyphenols (TPC = 459.6 mg GA/100 g).

The obtained AC and TPC results indicate that the size of the spice powders significantly impacted the antioxidant properties of the prepared extracts. The extracts obtained from the spice powder sieved by a smaller mesh size generally had higher levels of antioxidants. This suggests that antioxidant compounds in finer particles are exhibited to be more extractable by two commonly used solvents. The similar efficiency of antioxidant extraction for ethanol and an ethanol-water mixture can be explained by the theory that ethanol may weaken the bonds between polyphenolics-protein and polyphenolics-cellulose, releasing antioxidants from the studied spices [32]. Moreover, the higher peripheral velocity of the grinder and the longer exposure time of the ground spices appeared to be sufficient to recover more of the bioactive compounds from them in the prepared extracts. This supports the hypothesis that a higher peripheral velocity of the grinder could produce free radicals during the grinding process, which may act as stress signals and trigger stress responses in spices, causing the greater synthesis of antioxidants at more extended storage in contact with air, thus increasing their antioxidant potential [33]. In contrast, Ghasemzadeh et al. [34] demonstrated the negative effect of storage time on antioxidant properties. 

The TPC results of the ground ginger rhizome and nutmeg extracts were in agreement with some previously published data (TPC = 101.6–2314.0 mg GA/100 g and 49.82–268.0 mg GA/100 g for ginger and nutmeg, respectively) [35,36,37,38,39,40]. However, these authors [36,37,39] found significantly lower radical scavenging capacities of ginger and nutmeg against DPPH radical (0.922 mmol TE/100 g and 0.844 mmol TE/100 g, respectively) and ABTS radical cations (0.348 mmol TE/100 g and 0.455–19.1 mmol TE/100 g, respectively) compared with DPPH and ABTS values for the studied extracts of the two spice powders. Discrepancies in the antioxidant properties of spices can be explained by the influences of genetic, agronomic, and environmental factors, as well as different solvents and techniques used in the preparation of spice samples, which would affect the antioxidant contents.

This study, similar to other works [35,36], demonstrated that AC and TPC in ginger rhizome and nutmeg have a significant and positive correlation. Therefore, regression analysis was performed for the correlations among the antioxidant properties of the ethanol and ethanol-water extracts of both spices, as determined by the proposed DPPH, ABTS, and F–C methods. The calculated correlation coefficients (r = 0.5122–0.7905, *p* = 0.0000001–0.0038) suggest that there are significant relationships among the antioxidant potential results for extracts obtained using two different solvents. The obtained results of AC indicated that there are significant, positive correlations between the DPPH and ABTS values for the ethanol (r = 0.7905, *p* = 0.0000001) and ethanol-water (r = 0.6720, *p* = 0.00005) extracts of the investigated spices. These high r values demonstrated that the antioxidant compounds present in spice extracts were capable of scavenging DPPH radical and ABTS radical cations. However, lower correlation coefficients were calculated between DPPH–F–C methods for ethanol (r = 0.5593, *p* = 0.0013) and ethanol-water (r = 0.5122, *p* = 0.0038) extracts of spices. This suggests that the DPPH method brings an important limitation to the determination of hydrophilic antioxidants such as polyphenols [41]. However, significant linear correlations between the ABTS and F–C methods for the studied spice extracts were found (r = 0.7274, *p* = 0.000005 and r = 0.7077, *p* = 0.000012 for the ethanol and ethanol-water extracts, respectively). This can be explained by the fact that the ABTS radical cation enables the simultaneous determination of hydrophilic and lipophilic antioxidants present in the prepared extracts of spices [41].

### 2.2. Model Fitting and Data Analysis Using Response Surface Methodology

The RSM was applied to obtain the optimal parameters of the grinding process and storage time of ground ginger rhizome and nutmeg. The regression coefficients of the polynomial Equation (1) were calculated using experimental values, including DPPH, ABTS, and TPC in the ethanol and ethanol-water extracts of ginger rhizome and nutmeg (Table 1 and Table 2), and the generated equations were used in the prediction of the response values of the antioxidant properties of the investigated extracts of spices. The final partial cubic model (PCM) for the preparation of ginger rhizome and nutmeg before the extraction of antioxidants could be represented by the polynomial equations suggested using ANOVA as listed in Table 3.

The ANOVA results (Table 4) for the developed partial cubic model (PCM, Equation (1)) of all the three dependent variables (DPPH, ABTS, and TPC in the ethanol and ethanol-water extracts of ginger rhizome and nutmeg) revealed that the models were adequate with a desirable coefficient of multiple determination (R^2^) and adjusted R^2^.

As can be seen, the R^2^ values for these response variables were higher than 0.8, indicating that the proposed regression models were of goodness of fit [42]. However, low values of adjusted R^2^ = 0.6868 and 0.7267 for the responses of DPPH and TPC determined by the F–C method for the nutmeg ethanol extract did not indicate a close agreement between the experimental and predicted results.

The PCM model adequacy was tested using the lack-of-fit Fisher’s test (F-test), which was insignificant for *p* > 0.05 (Table 4). The ANOVA results of DPPH, ABTS, and TPC for the ginger rhizome and nutmeg ethanol and ethanol-water extracts revealed an insignificant lack of fit (F values = 0.044–18.3, *p* > 0.05). Therefore, these models were adequate for prediction within the range of variables employed. In addition, high F-values (53.7–361.1 for the DPPH and ABTS of both extracts and TPC in the ethanol extract of ginger rhizome, as well as 68.5–218.9 for the ABTS of the ethanol and ethanol-water extracts and TPC in the ethanol-water extract of nutmeg) demonstrated that the empirical models were significant with low probability values (*p* < 0.05). This suggests that the proposed mathematical models are valid and convenient for predicting the antioxidant properties of ginger rhizome (except TPC in ethanol-water extract) and nutmeg (except DPPH for both studied extracts and TPC in ethanol extract) as determined by the DPPH, ABTS, and F–C methods under any combinations of variables (the preparation conditions of spices for extraction process). Obviously, the low F-values (16.1 and 18.3 for TPC in the ethanol extract of nutmeg and the ethanol-water extract of ginger rhizome, respectively, as well as 26.2 and 26.9 for the DPPH of both extracts of nutmeg) and *p*-values > 0.05 suggest that the models’ predictions of the total amounts of polyphenols and antioxidants able to quench the DPPH radical in the prepared extracts of nutmeg were insignificant.

Therefore, only the linear parameter of the mesh size of the grinder sieve (X_1_) and two quadratic terms of the peripheral velocity of the grinding wheels $(X_{2}^{2}$) and the storage time of the ground spices $\left(X_{3}^{2}\right)$ had significant effects on DPPH values of the studied extracts of nutmeg (F = 19.2–166.7, *p* = 0.0059–0.048) and TPC in the ethanol (F = 38.1–50.4, *p* = 0.019–0.025) and ethanol-water (F = 39.7–73.6, *p* = 0.013–0.024) extracts of nutmeg and ginger rhizome, respectively. Unexpectedly, the same parameters showed significance on the DPPH of the ethanol extract of ginger rhizome. However, a high corresponding determination coefficient, R^2^ = 0.9983, adjusted as R^2^ = 0.9923 and a significant F value = 125.3 of this model indicate a good relationship between experimental and predicted values (Table 4). Moreover, one linear term (X_1_) and all quadratic terms $\left(X_{1}^{2},{\;X}_{2}^{2},{\;X}_{3}^{2}\right)$ showed significance (F = 19.8–331.3, *p* = 0.0030–0.047) on the ABTS of ethanol ginger rhizome extract. However, all linear terms (X_1_, X_2_, X_3_), two quadratic parameters ($X_{1}^{2}$ and $X_{2}^{2}$), and interactions between these independent variables ($X_{1}^{2}\times {\;X}_{2}$) exhibited significant effects (F = 18.9–874.4, *p* = 0.0011–0.049) on the ABTS of the ethanol-water ginger rhizome extract. On the contrary, this interaction ($X_{1}^{2}\times {\;X}_{2}$) and storage time of ground ginger rhizome ($X_{3}$ and $X_{3}^{2}$) caused insignificant effects on the DPPH of the ethanol-water extract (F = 0.083–1.53, *p* = 0.341–0.800) and TPC in ethanol extract (F = 0.645–14.3, *p* = 0.063–0.506).

In addition, the linear parameter of the peripheral velocity of the grinding wheels (X_2_), the quadratic term of the mesh size of the grinder sieve ($X_{1}^{2}$), and the interactions between X_2_ × X_3_ and $X_{1}^{2}\times {\;X}_{3}$ had no significant effects on the TPC in the prepared ethanol-water extract of nutmeg (F = 0.037–18.1, *p* = 0.051–0.603). The similar terms, all linear (X_1_, X_2_, X_3_), two quadratic parameters $\left(X_{2}^{2},{\;X}_{3}^{2}\right)$, and interaction (X_1_ × X_3_) or ($X_{1}^{2}\times {\;X}_{2}$) indicated notable significant effects (F = 32.9–1418.9, *p* = 0.0007–0.029) on the ABTS of both investigated extracts of nutmeg.

### 2.3. Analysis of Response Surfaces

**Figure 1:**
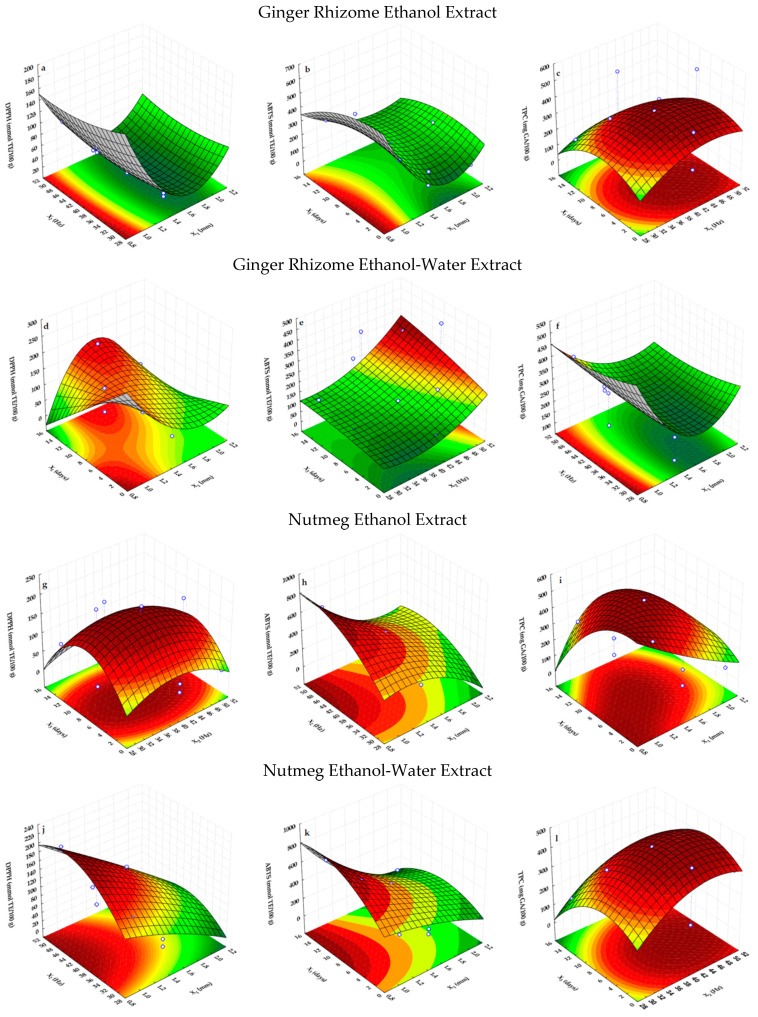
Response surfaces and contour plots showing the interactive influence of the mesh size of the grinder sieve (X_1_), the peripheral velocity of grinding wheels (X_2_) and the storage time of the ground spices (X_3_) on their antioxidant capacity determined by the DPPH (**a**,**d**,**g**,**j**), ABTS (**b**,**e**,**h**,**k**), and F–C (**c**,**f**,**i**,**l**) methods.

The regression equations, predicting the effects of the mesh size of the grinder sieve (X_1_), the peripheral velocity of grinding wheels (X_2_), and the storage time of the ground spices (X_3_), were explained by 3D response surface plots. The selected response surface plots were generated by plotting two factors over their respective ranges, while the third factor was kept at a constant value: 1.4 mm, 40 Hz, and 7 days for the X_1_, X_2_ and X_3_ independent variables, respectively (Figure 1).

It can be noted that the shapes of the response surfaces for DPPH, ABTS and TPC determined by the F–C method for the ethanol and ethanol-water extracts of the two studied spices were different from each other (Figure 1). However, the parabolic shapes of these surfaces indicate that the quadratic terms of independent variables were significant. The mesh size of the grinder sieve displayed negative linear (X_1_) and quadratic ($X_{1}^{2}$) effects on the antioxidant potential of the ethanol and ethanol-water extracts of ginger rhizome. Therefore, the response surfaces and contour plots of the DPPH and ABTS (Figure 1a,b) of the ethanol extract and TPC in the ethanol-water extract (Figure 1f) of this spice depicted a maximum at the lowest X_1_ (1.0 mm) and X_2_ (30 Hz) or an intermediate X_3_ (7 days). However, using a higher peripheral velocity of the grinding wheels, after 7 days of storage, the ethanol extract of ground ginger rhizome revealed the highest total polyphenols content (Figure 1c). The negative value of the linear term of the mesh size of the grinder sieve (X_1_) increased the DPPH of the ginger rhizome ethanol-water extract along with a decrease in the X_1_ independent variable (Figure 1d). However, the significant positive interaction between the corresponding variables (X_1_ × X_3_) and $X_{1}^{2}$ increased the DPPH of this extract prepared by ginger rhizome after using an intermediate mesh size of the grinder sieve size (X_1_ = 1.4 mm) for the longest storage time (X_3_ = 14 days). Moreover, the significant positive linear effect of X_2_ and X_3_ on the ABTS of the ginger rhizome ethanol-water extract resulted in the enhancement of its ability to scavenge the ABTS radical cation along with an increase in the peripheral velocity of the grinding wheels and the storage time of this spice (Figure 1e).

The parabolic shape of the response surface for the DPPH of the prepared nutmeg extracts (Figure 1g,j) was caused by the positive values of the quadratic terms of the peripheral velocity of the grinding wheels $\left(X_{2}^{2}\right)$ and the storage time of the ground nutmeg $\left(X_{3}^{2}\right)$. Therefore, the response surface and contour plot of the DPPH of the ethanol extract of nutmeg (Figure 1g) was the maximum at an intermediate peripheral velocity of the grinding wheels (40 Hz) after 7 days of storage. On the other hand, the negative linear effect of the mesh size of the grinder sieve (X_1_) with the quadratic term of the peripheral velocity of the grinding wheels used grinder $\left(X_{2}^{2}\right)$ showed that an increase in X_1_ decreased the DPPH results of the ethanol-water nutmeg extract, which is related to a consistently better extraction efficiency of antioxidants from samples ground with smaller mesh sizes of grinder sieve and higher peripheral velocity of grinding wheels (Figure 1j). The elliptical contours of the ABTS plots of the two nutmeg extracts confirm that there was an interaction between the independent variables (Figure 1h,k). It was observed that the ABTS results of both nutmeg extracts were raised with an increase in the peripheral velocity of the grinding wheels (X_2_) and the storage time (X_3_). Thus, the highest ABTS had extracts prepared from nutmeg ground using a higher peripheral velocity of the grinder (40–50 Hz), sieved through the lowest mesh (1.0 mm) after storage for a longer time (7–14 days) (Table 1 and Table 2). Moreover, the significant negative linear effect of X_1_ and the positive quadratic impact of X_3_ on the TPC in the ethanol extract of nutmeg resulted in an enhancement of the polyphenols concentration with the prolongation of the storage time of the nutmeg sieved using a smaller mesh size of the grinder sieve (Figure 1i). At a fixed mesh size (X_1_), the positive quadratic effects of the peripheral velocity of the grinding wheels $\left(X_{2}^{2}\right)$ and the storage time $\left(X_{3}^{2}\right)$ became more significant as their gradual increase enhanced the TPC in the ethanol-water extract of nutmeg (Figure 1l).

### 2.4. Verification of the Optimal Extraction Models

This study investigated the optimal conditions of spice preparation (the mesh size of the grinder sieve, the peripheral velocity of the grinding wheels, and the storage time) for producing the suitable antioxidant properties of the ethanol and ethanol-water extracts of ginger rhizome and nutmeg as determined by the DPPH, ABTS, and F–C methods. The optimal conditions to maximize the antioxidant potential of the two spices predicted by the response surface methodology are summarized in Table 5.

The optimal sample preparation conditions for antioxidant extraction were found to be dependent on the spice source and a solvent. As can be seen for the two spices, the best AC results were achieved for samples ground at 40 to 50 Hz of the peripheral velocity of the grinding wheels, sieved by 1.0 to 2.0 mm of the mesh size of the grinder sieve, and stored for a variable time of 1–9 days.

The predicted DPPH, ABTS, and TPC values for each extract of both spices at the optimal conditions were experimentally validated to verify the reliability of the optimization results. The experimental values of the antioxidant properties of the ethanol and ethanol-water extracts of ginger rhizome and nutmeg obtained under these optimized conditions were found to be close to the predicted values (Table 5). These results indicate that the Box–Behnken models successfully optimized the conditions of spice preparation for the increased yield of antioxidant extraction and the enhancement of antioxidant potential with an accurate and reliable prediction.

### 2.5. Composition of Phenolic Acids in Ginger Rhizome and Nutmeg Extracts Obtained Using Optimal Grinding Parameters and Storage Time

The spice extracts that were obtained by the optimum grinding process and storage time were analyzed to identify and quantify phenolic acids, and the results are presented in Table 6. In general, significantly (*p* ≤ 0.05) higher contents of phenolic acids were extracted from both ginger rhizome and nutmeg using pure ethanol (26 and 30% more in total, respectively, compared to ethanol-water extraction). Furthermore, the ginger rhizome extracts had a higher phenolic acid content (460.42 and 364.79 μg/100 mL in the ethanol and ethanol-water extracts, respectively) than the nutmeg extracts (300.52 and 199.86 μg/100 mL total in the ethanol and ethanol-water extracts, respectively).

Eleven phenolic acids were identified in the studied extracts. Regardless of the extractant used, vanillic acid was the main phenolic acid in the ginger rhizome extracts (up to 25%), while protocatechuic acid was the most abundant in the nutmeg extracts (up to 38%). The ethanol extract of ginger rhizome also contained higher amounts of *p*-OH-benzoic, ellagic, ferulic, and gallic acids, and lower amounts of salicylic, *p*-coumaric, protocatechuic, syringic, sinapic, and caffeic acids. In contrast, in the ethanol-water extract obtained from this spice, caffeic acid was not detected. The ethanol-water extract of ginger rhizome was also found to be richer in *p*-coumaric, protocatechuic, and sinapic acids compared to the ethanol extract. In nutmeg extracts, caffeic and ellagic acids were below detectable levels. In addition to protocatechuic acid, vanillic, ferulic, sinapic, and *p*-OH-benzoic (in ethanol-water extract) acids were found in higher amounts in these extracts, while the other acids were minor phenolic compounds in these extracts (up to 6.5% of total phenolic acids).

Variation in the composition of phenolic compounds in extracts obtained using different solvents has also been demonstrated in other studies. For example, Ghafoor et al. [11] showed that gallic acid, protocatechuic acid, catechin, and 1,2-dihydroxybenzene were the key phenolic constituents in the methanol-water extracts from ginger rhizome. In turn, Tohma et al. [43] detected pyrogallol, *p*-OH-benzoic acid, ferulic acid, vanillin, *p*-coumaric acid, gallic acid, and caffeic acid in ethanol extract obtained from this spice.

These various levels of phenolic acids from the same spice as a result of different solvents used for the extraction might be due to their properties, mainly hydrophobicity. Muzolf-Panek and Stuper-Szablewska [44] observed that spices richer in phenolic compounds with higher hydrophobicity (e.g., rosemary, clove) were characterized by the highest level of TPC in ethanol extract, while spices with a higher content of phenolic compounds with a relatively low hydrophobicity (e.g., caraway) showed the highest TPC values in water and aqueous extracts. Similarly, in our work, ethanol extracts contained a generally higher amount of more hydrophobic phenolic acids (e.g., *p*-OH-benzoic, vanillic, ferulic, sinapic acids) compared to ethanol-water extracts.

## 3. Materials and Methods

### 3.1. Materials

Fresh ginger (*Zingiber officinale* Roscoe) rhizome originating from Niger was purchased from Medium Company (Kalisz, Poland). The material was cleaned from the roots and outer cork layer, washed, dried, and sliced. Dried ginger root served as a research material. Nutmeg (*Myristica fragrans*) originating from Indonesia and was purchased from Medium Company. The formed dried, sorted nuclei of the nutmeg were used in the study. The materials were kept in a cool and dry place before being ground in a paper bag with a polyethylene (PE) liner.

### 3.2. Grinding Process and Storage Conditions

The ginger rhizome and nutmeg were initially shredded (universal shredder RU/S, Coffee Service Sp. z o.o., Warsaw, Poland), transferred to a grinder (universal grinder MUCS/S 800 DC, Coffee Service Sp. z o.o.), and ground in it according to the assumed parameters. The grinding process was carried out in the Confectionery Factory (Fabryka Cukiernicza Kopernik S.A., Toruń, Poland). Both spices were ground using grinder sieves with diameters of 1.0, 1.4, and 2.0 mm. The grinding proceeded at three peripheral velocities of grinding represented by the grinding motor inverter setting, at 30, 40, and 50 Hz. Furthermore, three different time frames between grinding and sample extraction were applied immediately after grinding (0 days) and 7 and 14 days after grinding. The ground spices were stored in a PE zip bag at 20 °C without light prior to analysis.

### 3.3. Preparation of Ethanol and Ethanol-Water Extracts

The ground spices (0.1000 ± 0.0001 g), prepared according to the Box–Behnken plan, were weighed into the test tubes with the screw caps, and then 5 mL of ethanol (Merck Life Science Sp. z o.o., Poznań, Poland) or a mixture of ethanol and water (1:1) was added. A suspension was formed by vigorously shaking the test tube. The suspension was extracted at 80 °C for 30 min in an ultrasonic bath (5200DTD; Chemland, Stargard Szczeciński, Poland). After extraction, the samples were centrifuged for 10 min in a laboratory centrifuge at 4500 rpm (MPW-54, Chemland), the extracts were separated from the spices, and the extractions were repeated by adding 5 mL of ethanol or ethanol-water mixture to the same sample.

### 3.4. Antioxidant Properties Determination

The AC and TPC in ethanol and ethanol-water extracts of two ground spices were determined by using the spectrophotometric DPPH, ABTS, and F–C methods, respectively, according to previously reported protocols [45]. The AC results were expressed as mmol Trolox equivalents (TE) per 100 g of sample, while the TPC values were expressed as mg gallic acid (GA) equivalents per 100 g of sample. All the reagents used in these methods were of analytical grade and were purchased from Merck Life Science Sp. z o.o. (Poznań, Poland): 2,2-diphenyl-1-picrylhydrazyl radical (DPPH), 2,2′ azino-bis (3-ethylbenzothiazoline-6-sulphonic acid) diammonium salt (ABTS), Folin–Ciocalteu’s phenol reagent (F–C reagent, 2 N), Trolox (6-hydroxy-2,5,7,8-tetramethylchromane-2-carboxylic acid) (TE, 97%), and gallic acid (3,4,5-trihydroxybenzoic acid) (GA, 98%). The resulting absorbance of each obtained solution was measured in five repetitions using a Hitachi U-2900 UV-VIS spectrophotometer (Tokyo, Japan) in a 1 cm glass cell. The AC and TPC results were calculated based on the standard curves: %DPPH = (782.10 ± 5.74)c_TE_ + (4.03 ± 0.40), %ABTS = (405.39 ± 3.40)c_TE_ + (10.38 ± 0.30), and TPC = (0.1034 ± 0.0025)c_GA_ + (0.0814 ± 0.0147) prepared for the working solutions in the concentration ranges of 0.02 and 0.10 μmol TE/mL, 0.01 and 0.15 μmol TE/mL, and 0.35–10.51 μg GA/mL, respectively.

### 3.5. Experimental Design and Mathematical Model

An experimental design based on the chemometric approach was preferred to reduce the number of experiments and to consider the interaction between the variables. The Box–Behnken experimental design based on 15 runs in RSM was selected for numerical optimization to achieve the best response values. A three-factor, three-level response surface test was designed with the mesh size of the grinder sieve (X_1_, mm), the peripheral velocity of the grinding wheels (X_2_, Hz), and the storage time of the ground spices (X_3_, days) as independent variables, and the antioxidant properties of ginger rhizome and nutmeg, determined by three analytical methods, DPPH, ABTS, and F–C, as response values. The independent variables for the grinding process and storage time of examined spices are listed in Table 1 and Table 2, where the experimental range and levels are specified (low, medium, and high denoted as −1, 0, and 1, respectively). The factor levels were fixed based on the preliminary experiment trials.

The effects of these three independent variables on the antioxidant properties of both studied spices can be approximated using the following partial cubic model (PCM) as shown in Equation (1):

(1) $$Y_{n}=\beta_{0}+\beta_{1}\times X_{1}+\beta_{2}\times X_{2}+\beta_{3}\times X_{3}+\beta_{11}\times X_{1}^{2}+\beta_{22}\times X_{2}^{2}+\beta_{33}\times X_{3}^{2}+\beta_{12}\times X_{1}\times X_{2}+\beta_{13}\times X_{1}\times X_{3}\;+\beta_{23}\times X_{2}\times X_{3}+\beta_{112}\times X_{1}^{2}\times X_{2}+\beta_{113}\times X_{1}^{2}\times X_{3}$$

where: Y_n_ is one of the three predicted responses, X_1_, X_2_ and X_3_ represent the independent variables, β_0_ is the constant, β_1_, β_2_, and β_3_ are the linear-term coefficients, β_11_, β_22_, and β_33_ are the quadratic-term coefficients, and β_12_, β_13_, β_23_, β_112_, and β_113_ are the cross-term coefficients.

The polynomial Equation (1) visualizes the relationship between the response and the experimental levels of each factor and deduces the optimum conditions from the response surface and contour plots. The coefficient of determination (R^2^) and adjusted coefficient of determination (adjusted R^2^) were used to evaluate the accuracy and general ability of the polynomial regression models. The significance of the independent variables, their interactions, and regression coefficients was tested by an analysis of variance (ANOVA) for each response. A lack-of-fit analysis was applied to determine the variance and adequacy of the model’s results that were fitted.

### 3.6. Determination of Phenolic Acids

The phenolic acids were determined chromatographically. Briefly, 20 mL of extract was evaporated to dryness at temperatures below 50 °C in an R-210-type Büchi vacuum evaporator (Büchi Labortechnik, Flawil, Switzerland). The residue was dissolved in 20 mL of deionized water (HLP 5 deionizer, Hydrolab, Gdańsk, Poland), acidified to pH 2 with 6 M hydrochloric acid (POCH, Gliwice, Poland), and 0.2 mL of 3,5-dichloro-2-hydroxybenzoic solution (1 mg/1 mL) in diethyl ether (Chempur, Piekary Śląskie, Poland) as an internal standard was added. Then, phenolic acids were extracted 5 times with 20 mL of diethyl ether, and the collected extracts were evaporated in a vacuum evaporator (Büchi Labortechnik, type R-210). The dry extract was re-dissolved in 2 mL of methanol and subjected to chromatographic separation. Phenolic acid UPLC analysis was performed on an Agilent 1290 Infinity system coupled with a 6470 triple quadrupole mass spectrometer (Agilent Technologies, Santa Clara, CA, USA) with an electrospray ionization (ESI) source. Compounds were separated using a Synergi Fusion-RP column (100 × 2 mm, 2.8 μm, Phenomenex, Torrance, CA, USA) with the temperature set at 20 °C. A gradient elution program was employed, using two elution solvents: solvent A (water/formic acid; 99.9/0.1, *v*/*v*) and solvent B (acetonitrile/formic acid; 99.9/0.1, *v*/*v*). Chromatography-grade acetonitrile was purchased from Sigma-Aldrich (St. Louis, MO, USA, supplier Poznań, Poland), and analytical-grade formic acid was purchased from Chempur, Karlsruhe, Germany. The flow rate was 0.4 mL/min with a gradient elution program as follows: 0–1 min 97% A; 1–8 min, 97–60% A; 8–10 min, 60–40% A; 10–11 min, 40–97% A and was stable until 15 min. Mass spectrometry data were obtained by an electrospray ionization (ESI) source in negative ionization mode. Source conditions were: drying gas temperature of 350 °C, drying gas flow of 10 L/min, nebulizer pressure of 30 psi, sheath gas temperature of 300 °C, sheath gas flow of 11 L/min, and capillary voltage of 3500 V. Specific MRM mode parameters for the targeted compounds were optimized through the Agilent optimizer software (Mass Hunter Optimizer), including MRM transitions, collision energy, fragmentor voltage, dwell time, and cell accelerator voltage. The selected parameters for phenolic acids are scheduled in Table 7. All standards of phenolic acids (declared purity of >97%) were purchased from Sigma-Aldrich.

The contents of phenolic acids were determined from the calibration curves of reference standards. The internal standard used aided in better quantification to decide whether the compound recovery is complete. The calibration parameters of phenolic acids with their standard deviations, regression coefficient (R^2^), limit of detection (LOD), and limit of quantification (LOQ) values are given in Table 8.

### 3.7. Statistical Analysis

All experimental runs were conducted five or three times and are presented as the mean ± standard deviation (SD). Tukey’s test was performed to analyze significant differences (*p* ≤ 0.05) between the obtained results of the DPPH, ABTS, TPC, and individual phenolic compounds in both investigated extracts of ginger rhizome and nutmeg.

The Pearson correlation analysis was performed to establish the correlations between the three analytical methods used for the determination of the antioxidant properties of spice extracts prepared using different conditions of the grinding process and two solvents.

The Statistica 8.0 software (StatSoft, Tulsa, OK, USA) was utilized for the statistical analysis, the design of the experiment, the construction of the response surface contour plots, and the calculation of the optimum conditions.

## 4. Conclusions

In the current study, the grinding parameters and storage time of the ground species influenced the antioxidant properties of ethanol and ethanol-water extracts. It was observed that the mesh size had the most significant negative effect on the antioxidant properties of all studied extracts of ginger rhizome and ethanol-water extracts of nutmeg. However, the storage time of ground nutmeg and the peripheral velocity of grinding were more effective independent variables on the DPPH and TPC results of its ethanol extracts, respectively. Furthermore, the experimental results agreed well with the predicted values, indicating that the Box–Behnken model can be successfully used to optimize the conditions of spice preparation before the extraction of antioxidants for food applications. Besides the spice-preparing parameters, the polarity of the solvent used as an extractant is also important. A comparison of the antioxidant properties of the extracts prepared with two commonly used solvents indicated that the ethanol-water mixture increased the extraction of the total phenols from ginger rhizome, while for the extraction of total phenols from nutmeg, ethanol is more suitable. However, ethanol, regardless of the specie type, proved to be a more efficient solvent for the extraction of phenolic acids. Furthermore, the ginger rhizome extracts had a higher phenolic acid content than the nutmeg extracts. These findings could help the food industry to produce cost-effective products containing spices rich in antioxidant compounds. Both types of spices are important ingredients in gingerbread recipes, so especially this way of using optimization in their preparation before adding them to the dough seems advisable.

## Figures and Tables

**Table 1:** Three-level Box–Behnken design with experimental ($\bar{x}$ ± *SD*) and predicted results for the antioxidant properties of ginger rhizome.

Exp.	Independent Variables			Dependent Variables					
	X_1_ (mm)	X_2_ (Hz)	X_3_ (Days)	DPPH Method (mmol TE/100 g)		ABTS Method (mmol TE/100 g)		F–C Method (mg GA/100 g)	
				Experimental	Predicted	Experimental	Predicted	Experimental	Predicted
Extraction with Ethanol									
1	2.0 (1)	40 (0)	0 (−1)	22.5 ± 0.1 ^a^	22.3	95.3 ± 1.9 ^a^	92.4	153.9 ± 3.2 ^b,c^	149.6
2	2.0 (1)	40 (0)	14 (1)	29.6 ± 0.3 ^c^	29.4	157.3 ± 2.8 ^b^	154.4	151.5 ± 2.4 ^a,b^	147.2
3	2.0 (1)	50 (1)	7 (0)	42.2 ± 0.9 ^f^	42.5	229.1 ± 0.9 ^g^	232.0	162.7 ± 1.6 ^c,d^	167.0
4	2.0 (1)	30 (−1)	7 (0)	38.5 ± 0.2 ^e^	38.8	198.2 ± 0.9 ^d,e^	201.1	202.7 ± 1.9 ^f^	207.0
5	1.4 (0)	50 (1)	0 (−1)	23.1 ± 0.5 ^a^	23.1	96.9 ± 2.3 ^a^	96.9	278.5 ± 1.5 ^g^	278.5
6	1.4 (0)	50 (1)	14 (1)	37.6 ± 0.6 ^e^	37.6	184.0 ± 4.0 ^c^	184.0	178.5 ± 2.6 ^e^	178.5
7	1.4 (0)	30 (−1)	0 (−1)	26.9 ± 0.3 ^b^	26.9	196.0 ± 2.0 ^d^	196.0	165.3 ± 2.8 ^d^	165.3
8	1.4 (0)	30 (−1)	14 (1)	33.1 ± 0.3 ^d^	33.1	262.0 ± 2.0 ^h^	262.0	152.9 ± 2.6 ^a,b^	152.9
9	1.4 (0)	40 (0)	7 (0)	31.9 ± 0.6 ^d^	28.8	209.5 ± 1.6 ^f^	197.9	355.9 ± 4.8 ^h,i^	355.8
10	1.0 (−1)	40 (0)	0 (−1)	86.4 ± 0.7 ^g^	86.7	378.0 ± 8.0 ^j^	380.9	383.3 ± 4.1 ^j^	387.6
11	1.0 (−1)	40 (0)	14 (1)	95.0 ± 1.2 ^h^	95.3	308.0 ± 5.0 ^i^	310.9	464.1 ± 4.0 ^l^	468.4
12	1.0 (−1)	50 (1)	7 (0)	99.0 ± 1.2 ^i^	98.8	430.0 ± 5.0 ^k^	427.1	507.4 ± 11.5 ^m^	503.1
13	1.0 (−1)	30 (−1)	7 (0)	113.0 ± 2.0 ^j^	112.8	454.0 ± 9.0 ^l^	451.1	434.5 ± 2.7 ^k^	430.2
14	1.4 (0)	40 (0)	7 (0)	29.4 ± 0.5 ^c^	28.8	206.1 ± 1.9 ^e,f^	197.9	348.0 ± 3.2 ^h^	355.8
15	1.4 (0)	40 (0)	7 (0)	25.2 ± 0.2 ^b^	28.8	178.0 ± 4.0 ^c^	197.9	363.4 ± 4.4 ^i^	355.8
Extraction with Ethanol-Water									
1	2.0 (1)	40 (0)	0 (−1)	52.9 ± 1.3 ^c^	45.6	99.3 ± 1.0 ^b^	79.3	146.5 ± 2.0 ^b^	131.0
2	2.0 (1)	40 (0)	14 (1)	67.1 ± 0.9 ^d^	59.8	108.6 ± 1.7 ^b^	88.6	158.6 ± 2.9 ^c^	143.1
3	2.0 (1)	50 (1)	7 (0)	43.6 ± 0.9 ^b^	50.9	127.0 ± 2.8 ^c^	147.0	228.0 ± 2.4 ^g^	243.6
4	2.0 (1)	30 (−1)	7 (0)	26.4 ± 0.6 ^a^	33.7	101.9 ± 2.5 ^b^	121.9	211.6 ± 1.8 ^f^	227.2
5	1.4 (0)	50 (1)	0 (−1)	85.9 ± 1.8 ^f^	85.9	171.0 ± 4.1 ^e^	171.0	199.8 ± 2.8 ^e^	199.8
6	1.4 (0)	50 (1)	14 (1)	189.9 ± 2.6 ^m^	189.9	284.8 ± 2.7 ^f^	284.8	181.1 ± 3.0 ^d^	181.1
7	1.4 (0)	30 (−1)	0 (−1)	54.0 ± 0.2 ^c^	54.0	80.4 ± 1.3 ^a^	80.4	81.0 ± 1.2 ^a^	81.0
8	1.4 (0)	30 (−1)	14 (1)	112.6 ± 4.7 ^h^	112.6	125.3 ± 2.3 ^c^	125.3	185.1 ± 2.2 ^d^	185.1
9	1.4 (0)	40 (0)	7 (0)	120.4 ± 0.9 ^i^	122.4	145.7 ± 1.8 ^d^	130.4	215.1 ± 1.6 ^f^	225.8
10	1.0 (−1)	40 (0)	0 (−1)	171.2 ± 3.7 ^l^	178.5	321.3 ± 6.6 ^g^	341.3	332.6 ± 2.8 ^i^	348.2
11	1.0 (−1)	40 (0)	14 (1)	78.7 ± 0.9 ^e^	86.0	367.0 ± 5.6 ^h^	387.0	189.0 ± 1.3 ^d^	204.6
12	1.0 (−1)	50 (1)	7 (0)	166.0 ± 0.7 ^k^	158.7	429.3 ± 7.7 ^i^	409.3	390.2 ± 8.1 ^j^	374.7
13	1.0 (−1)	30 (−1)	7 (0)	92.6 ± 1.8 ^g^	85.3	440.0 ± 9.2 ^j^	420.0	390.3 ± 8.7 ^j^	374.8
14	1.4 (0)	40 (0)	7 (0)	118.8 ± 1.8 ^i^	122.4	120.9 ± 2.2 ^c^	130.4	210.2 ± 4.0 ^f^	225.8
15	1.4 (0)	40 (0)	7 (0)	128.0 ± 2.1 ^j^	122.4	124.5 ± 3.0 ^c^	130.4	252.2 ± 4.4 ^h^	225.8

*n* = 5; $\bar{x}$ ± *SD*—mean value ± standard deviation; different letters (^a–m^) within the same column indicate significant differences (one-way ANOVA and Tukey’s test, *p* ≤ 0.05); coded levels are given in parentheses; X_1_—mesh size of grinder sieve, X_2_—peripheral velocity of grinding wheels, X_3_—storage time of ground spices.

**Table 2:** Three-level Box–Behnken design with experimental ($\bar{x}$ ± *SD*) and predicted results for the antioxidant properties of nutmeg.

Exp.	Independent Variables			Dependent Variables					
	X_1_ (mm)	X_2_ (Hz)	X_3_ (Days)	DPPH Method (mmol TE/100 g)		ABTS Method (mmol TE/100 g)		F–C Method (mg GA/100 g)	
				Experimental	Predicted	Experimental	Predicted	Experimental	Predicted
Extraction with Ethanol									
1	2.0 (1)	40 (0)	0 (−1)	29.8 ± 0.6 ^a^	7.4	71.9 ± 1.2 ^a^	66.7	125.9 ± 2.4 ^a^	163.8
2	2.0 (1)	40 (0)	14 (1)	42.1 ± 1.1 ^c^	19.7	163.0 ± 4.0 ^c^	157.8	191.1 ± 3.7 ^c^	229.0
3	2.0 (1)	50 (1)	7 (0)	60.7 ± 0.7 ^e^	83.1	206.0 ± 3.0 ^d^	211.2	246.3 ± 2.4 ^e^	208.4
4	2.0 (1)	30 (−1)	7 (0)	31.1 ± 0.6 ^a^	53.5	62.3 ± 1.5 ^a^	67.5	187.1 ± 3.2 ^c^	149.2
5	1.4 (0)	50 (1)	0 (−1)	35.3 ± 0.4 ^b^	35.3	143.7 ± 1.1 ^b^	143.7	243.1 ± 3.0 ^e^	243.1
6	1.4 (0)	50 (1)	14 (1)	41.2 ± 0.7 ^c^	41.2	327.0 ± 6.0 ^f^	327.0	320.5 ± 3.0 ^g^	320.5
7	1.4 (0)	30 (−1)	0 (−1)	53.7 ± 0.9 ^d^	53.7	154.0 ± 3.0 ^b,c^	154.0	144.6 ± 2.8 ^b^	144.6
8	1.4 (0)	30 (−1)	14 (1)	77.6 ± 0.7 ^f^	77.6	223.0 ± 3.0 ^e^	223.0	232.7 ± 1.5 ^d^	232.7
9	1.4 (0)	40 (0)	7 (0)	173.4 ± 1.4 ^i^	174.3	446.9 ± 10.2 ^i^	449.2	445.7 ± 2.9 ^i^	487.9
10	1.0 (−1)	40 (0)	0 (−1)	52.4 ± 0.6 ^d^	74.8	430.0 ± 10.0 ^h^	435.2	495.5 ± 6.6 ^j^	457.6
11	1.0 (−1)	40 (0)	14 (1)	140.5 ± 1.2 ^g^	162.9	506.0 ± 6.0 ^k^	511.2	315.9 ± 3.7 ^g^	278.0
12	1.0 (−1)	50 (1)	7 (0)	162.2 ± 2.6 ^h^	139.8	634.0 ± 14.0 ^l^	628.8	362.8 ± 5.7 ^h^	400.7
13	1.0 (−1)	30 (−1)	7 (0)	230.0 ± 5.0 ^k^	207.6	377.0 ± 7.0 ^g^	371.8	261.7 ± 4.7 ^f^	299.6
14	1.4 (0)	40 (0)	7 (0)	189.5 ± 1.9 ^j^	174.3	437.0 ± 9.0 ^h,i^	449.2	506.8 ± 7.0 ^k^	487.9
15	1.4 (0)	40 (0)	7 (0)	159.9 ± 1.4 ^h^	174.3	463.8 ± 2.4 ^j^	449.2	511.1 ± 9.7 ^k^	487.9
Extraction with Ethanol-Water									
1	2.0 (1)	40 (0)	0 (−1)	28.3 ± 0.9 ^a^	21.5	165.5 ± 3.8 ^b^	140.5	169.2 ± 3.3 ^b^	186.7
2	2.0 (1)	40 (0)	14 (1)	41.8 ± 2.0 ^b^	35.0	170.3 ± 3.1 ^b^	145.3	198.6 ± 2.3 ^c^	216.1
3	2.0 (1)	50 (1)	7 (0)	39.1 ± 0.6 ^b^	45.9	191.8 ± 4.2 ^c^	216.8	279.9 ± 4.4 ^e^	262.4
4	2.0 (1)	30 (−1)	7 (0)	33.4 ± 0.4 ^a,b^	40.2	147.5 ± 3.4 ^a^	172.5	233.3 ± 4.6 ^d^	215.8
5	1.4 (0)	50 (1)	0 (−1)	64.0 ± 0.7 ^d^	64.0	261.1 ± 5.8 ^e^	261.1	348.5 ± 2.8 ^g^	348.5
6	1.4 (0)	50 (1)	14 (1)	89.4 ± 1.4 ^e^	89.4	345.9 ± 7.9 ^f^	345.9	281.2 ± 4.9 ^e^	281.2
7	1.4 (0)	30 (−1)	0 (−1)	36.1 ± 0.3 ^a,b^	36.1	204.5 ± 4.4 ^c^	204.5	232.7 ± 5.1 ^d^	232.7
8	1.4 (0)	30 (−1)	14 (1)	53.1 ± 0.6 ^c^	53.1	238.1 ± 4.4 ^d^	238.1	148.2 ± 1.3 ^a^	148.2
9	1.4 (0)	40 (0)	7 (0)	143.5 ± 1.7 ^h^	149.9	458.5 ± 10.9 ^g^	460.8	443.7 ± 5.0 ^i^	427.5
10	1.0 (−1)	40 (0)	0 (−1)	108.7 ± 4.7 ^f^	115.5	354.2 ± 5.0 ^f^	379.2	459.6 ± 6.6 ^j^	442.1
11	1.0 (−1)	40 (0)	14 (1)	154.1 ± 3.0 ^i^	160.9	620.1 ± 12.1 ^j^	645.1	272.7 ± 2.6 ^e^	255.2
12	1.0 (−1)	50 (1)	7 (0)	186.9 ± 1.9 ^j^	180.1	657.4 ± 0.7 ^k^	632.4	312.9 ± 7.6 ^f^	330.4
13	1.0 (−1)	30 (−1)	7 (0)	132.9 ± 4.1 ^g^	126.1	520.2 ± 19.4 ^i^	495.2	424.8 ± 8.6 ^h^	442.3
14	1.4 (0)	40 (0)	7 (0)	163.8 ± 5.3 ^i^	149.9	481.6 ± 3.0 ^h^	460.8	419.1 ± 7.1 ^h^	427.5
15	1.4 (0)	40 (0)	7 (0)	142.4 ± 2.5 ^g,h^	149.9	442.3 ± 8.4 ^g^	460.8	419.6 ± 4.4 ^h^	427.5

*n* = 5; $\bar{x}$ ± *SD*—mean value ± standard deviation; different letters (^a–l^) within the same column indicate significant differences (one-way ANOVA and Tukey’s test, *p* ≤ 0.05); coded levels are given in parentheses; X_1_—mesh size of grinder sieve, X_2_—peripheral velocity of grinding wheels, X_3_—storage time of ground spices.

**Table 3:** Regression coefficients of the partial cubic model for the antioxidant properties of ginger rhizome and nutmeg extracts.

Term	Coefficient					
	DPPH Method (mmol TE/100 g)	ABTS Method (mmol TE/100 g)	F–C Method (mg GA/100 g)	DPPH Method (mmol TE/100 g)	ABTS Method (mmol TE/100 g)	F–C Method (mg GA/100 g)
	Ginger Rhizome Ethanol Extract			Ginger Rhizome Ethanol-Water Extract		
β_0_	839.4 *	1372.3	−52.4	351.5	4752.7 **	2748.2
β_1_	−831.1 *	−223.9	−1164.1	−666.8	−5288.8 **	−3247.7
β_2_	−11.3	−0.44	43.5 *	12.7	−88.9 *	−32.7
β_3_	0.06	−58.1	114.6 **	−98.6 **	−30.5	−54.5
β_11_	243.8 *	−58.1	384.0	215.4	1651.7 *	999.2
β_22_	0.08 *	0.40 *	−0.62 **	−0.11 *	0.43 *	0.17
β_33_	−0.14	−1.1 *	−2.1 **	−0.02	−0.16	−1.7 *
β_12_	5.4	−51.3	20.4	−0.51	77.7 *	32.6
β_13_	2.0	93.1	−98.6 *	124.7 **	40.4	121.9
β_23_	0.03	0.08	−0.31 *	0.16 *	0.25	−0.44
β_112_	−1.5	18.0	−8.7	−0.77	−25.3 *	−10.6
β_113_	−0.70	−27.9	30.9 *	−39.0 **	−14.4	−36.9
	Nutmeg Ethanol Extract			Nutmeg Ethanol−Water Extract		
β_0_	−314.9	−3969.4 **	−1468.2	−702.7	−1449.1	2880.1 *
β_1_	81.9	3555.8 *	−568.9	183.9	673.6	−4654.7 *
β_2_	18.6	168.4 **	111.9	35.7	76.3 *	−31.1
β_3_	65.1 *	−0.46	−93.1	26.0	126.9 *	−15.5
β_11_	−114.7	−1232.6 *	119.6	−60.4	−242.2	1360.8 *
β_22_	−0.34 *	−1.1 **	−1.4 *	−0.37 *	−0.73 *	−1.7 *
β_33_	−1.8 **	−2.7 **	−2.4 *	−1.1 *	−2.6 **	−2.2 **
β_12_	5.8	−108.7 *	3.4	−4.0	−15.8	116.0 *
β_13_	−43.7	40.5	164.3	−12.5	−110.0	37.3
β_23_	−0.06	0.41	−0.04	0.03	0.18	0.06
β_112_	−0.30	34.3 *	−1.8	0.54	3.7	−36.0 *
β_113_	12.8	−13.1	−48.9	3.4	30.4	−7.3

Significant at the * *p* < 0.05; ** *p* < 0.01.

**Table 4:** Analysis of variance (ANOVA) results for the responses: antioxidant properties of ginger rhizome and nutmeg extracts determined by DPPH, ABTS, and F–C methods.

Model Parameters	df	SS	MS	F Value	SS	MS	F Value
		Ginger Rhizome Ethanol Extract			Ginger Rhizome Ethanol-Water Extract		
		DPPH Method					
Regression	11	15,804.6	1436.8	125.3 *	31,255.0	2841.4	117.6 *
Residual	3	23.4	7.8		479.0	159.7	
Lack-of-fit	1	0.5	0.5	0.044	430.7	430.7	17.8
Pure error	2	22.9	11.5		48.3	24.2	
Total	14	15,828.0			31,734.0		
R^2^, Adjusted R^2^	0.9983, 0.9923				0.9859, 0.9341		
		ABTS Method					
Regression	11	176,690.3	16,062.8	53.7 *	242,400.4	22,036.4	122.7 *
Residual	3	665.7	221.9		3559.1	1186.4	
Lack-of-fit	1	67.9	67.9	0.23	3200.0	3200.0	17.8
Pure error	2	597.8	298.9		359.1	179.6	
Total	14	177,356.0			245,959.5		
R^2^, Adjusted R^2^	0.9960, 0.9814				0.9845, 0.9274		
		F–C Method					
Regression	11	235,560.5	21,414.6	361.1 **	106,111.1	9646.5	18.3
Residual	3	267.4	89.1		2989.2	996.4	
Lack-of-fit	1	148.8	148.8	2.5	1934.4	1934.4	3.7
Pure error	2	118.6	59.3		1054.8	527.4	
Total	14	235,827.9			109,100.3		
R^2^, Adjusted R^2^	0.9988, 0.9945				0.9713, 0.8661		
		Nutmeg Ethanol Extract			Nutmeg Ethanol-Water Extract		
		DPPH Method					
Regression	11	63,367.1	5760.6	26.2	42,957.5	3905.2	26.9
Residual	3	4462.3	1487.4		663.1	221.0	
Lack-of-fit	1	4023.1	4023.1	18.3	372.7	372.7	2.6
Pure error	2	439.2	219.6		290.4	145.2	
Total	14	67,829.4			43,620.6		
R^2^, Adjusted R^2^	0.9329, 0.6868				0.9847, 0.9286		
		ABTS Method					
Regression	11	44,2194.8	40,199.5	218.9 **	409,498.0	37227.1	95.4 *
Residual	3	583.6	194.5		5770.2	1923.4	
Lack-of-fit	1	216.3	216.3	1.2	4990.0	4990.0	12.8
Pure error	2	367.3	183.7		780.2	390.1	
Total	14	44,2778.4			415,268.2		
R^2^, Adjusted R^2^	0.9987, 0.9937				0.9859, 0.9340		
		F–C Method					
Regression	11	236,564.6	21,505.9	16.1	149,008.4	13,546.2	68.5 *
Residual	3	14,175.2	4725.1		2845.4	948.5	
Lack-of-fit	1	11,498.9	11498.9	8.6	2450.0	2450.0	12.4
Pure error	2	2676.3	1338.2		395.4	197.7	
Total	14	250,739.8			151,853.8		
R^2^, Adjusted R^2^	0.9414, 0.7267				0.9813, 0.9128		

df—degrees of freedom; SS—sum of square; MS—mean of square; R^2^—coefficient of determination; F value—F-statistic; * Significant at the probability value, *p* < 0.05 level; ** Significant at the *p* < 0.01 level.

**Table 5:** Predicted and experimental ($\bar{x}$ ± *SD*) values of the studied responses for the optimum conditions of spices preparation.

Response Variable	Optimum Conditions			Predicted Values	Experimental Values
	X_1_ [mm]	X_2_ [Hz]	X_3_ [Days]		
Ginger Rhizome Ethanol Extract					
DPPH (mmol TE/100 g)	2.0	43	9	30.0	34.5 ± 0.1
ABTS (mmol TE/100 g)				179.5	176.2 ± 2.5
TPC (mg GA/100 g)				222.6	216.0 ± 9.3
Ginger Rhizome Ethanol-Water Extract					
DPPH (mmol TE/100 g)	1.0	50	1	189.9	180.8 ± 4.3
ABTS (mmol TE/100 g)				365.5	361.4 ± 2.6
TPC (mg GA/100 g)				399.8	392.3 ± 8.6
Nutmeg Ethanol Extract					
DPPH (mmol TE/100 g)	1.0	41	7	204.4	209.8 ± 0.3
ABTS (mmol TE/100 g)				620.3	619.55 ± 2.8
TPC (mg GA/100 g)				486.1	482.9 ± 3.9
Nutmeg Ethanol-Water Extract					
DPPH (mmol TE/100 g)	1.0	40	7	188.3	185.3 ± 0.2
ABTS (mmol TE/100 g)				630.6	659.4 ± 2.3
TPC (mg GA/100 g)				459.5	453.2 ± 7.4

*n* = 5; $\bar{x}$ ± *SD*—mean value ± standard deviation.

**Table 6:** Content of phenolic acids ($\bar{x}$ ± *SD*) in ginger rhizome and nutmeg extracts obtained with the optimal grinding process and storage time.

Phenolic Compounds (μg/100 mL)	Ginger Rhizome Extract		Nutmeg Extract	
	Ethanol	Ethanol-Water	Ethanol	Ethanol-Water
Caffeic acid	6.71 ± 0.45	<LOD	<LOD	<LOD
Ellagic acid	62.58 ± 3.09 ^a^	26.90 ± 0.38 ^b^	<LOD	<LOD
Ferulic acid	59.99 ± 1.30 ^a^	49.45 ± 1.28 ^b^	34.64 ± 2.05 ^c^	22.47 ± 0.16 ^d^
Gallic acid	53.60 ± 1.86 ^a^	43.08 ± 4.43 ^b^	<LOD	<LOD
*p*-Coumaric acid	23.10 ± 1.15 ^b^	35.99 ± 0.14 ^a^	19.36 ± 0.63 ^c^	12.94 ± 0.15 ^d^
*p*-OH-Benzoic acid	65.08 ± 2.75 ^a^	39.91 ± 0.13 ^b^	38.21 ± 0.89 ^b^	12.38 ± 0.40 ^c^
Protocatechuic acid	19.70 ± 1.93 ^d^	26.44 ± 0.72 ^c^	100.87 ± 1.40 ^a^	75.91 ± 0.55 ^b^
Salicylic acid	26.83 ± 2.67 ^b^	35.29 ± 2.01 ^a^	12.58 ± 1.43 ^c^	11.11 ± 0.75 ^c^
Sinapic acid	13.66 ± 1.45 ^b,c^	12.58 ± 0.92 ^c^	25.14 ± 1.74 ^a^	15.24 ± 0.16 ^b^
Syringic acid	13.98 ± 1.58 ^b^	11.72 ± 0.18 ^c^	18.12 ± 0.73 ^a^	11.55 ± 0.39 ^c^
Vanillic acid	115.19 ± 5.18 ^a^	83.43 ± 0.70 ^b^	51.60 ± 0.19 ^c^	38.26 ± 0.71 ^d^

LOD—limit of detection; *n* = 3; $\bar{x}$ ± *SD*—mean value ± standard deviation; different letters (^a–d^) within the same line indicate significant differences (one-way ANOVA and Tukey’s test, *p* ≤ 0.05).

**Table 7:** Related MS data of investigated phenolic acids in the UPLC analysis.

Phenolic Compounds	[M-H]- (*m*/*z*)	Product Ion (*m*/*z*)	Dwell	Fragmentor (V)	Collision Energy (V)	Cell Accelerator (V)
3,5-Dichloro-2-OH-benzoic acid	207	163	40	104	16	7
Caffeic acid	179	135.1	40	104	16	7
Chlorogenic acid	353.1	191.1	40	104	12	7
Ellagic acid	301	300.1	40	168	36	7
Ferulic acid	193.1	134.1	40	104	16	7
Gallic acid	169	125.1	40	104	12	7
*p*-Coumaric acid	163	119.1	40	72	16	7
Protocatechuic acid	153.1	109.1	40	104	12	7
*p*-OH-Benzoic acid	137	93.1	40	72	16	7
Salicylic acid	137.1	93.1	40	72	16	7
Sinapic acid	223.1	208.1	40	104	12	7
Syringic acid	197	182.1	40	104	12	7
Vanillic acid	167	152.1	40	72	12	7

**Table 8:** The calibration parameters of phenolic acids using rate of peak normalizations, with their determination coefficient, LOD, and LOQ values.

Phenolic Compounds	Equation of Linear Regression	R^2^	LOD	LOQ
3,5-Dichloro-2-OH-benzoic acid	y = 3061x + 40,191	0.959	0.118	0.392
Caffeic acid	y = 15,829x + 56,299	0.995	0.038	0.127
Chlorogenic acid	y = 12,320x + 25492	0.998	0.171	0.570
Ellagic acid	y = 1143x + 2849	0.995	0.215	0.717
Ferulic acid	y = 2442x + 2696	0.999	0.159	0.528
Gallic acid	y = 6702x + 61,152	0.978	0.549	1.830
*p*-Coumaric acid	y = 11,203x + 86,477	0.983	0.138	0.460
Protocatechuic acid	y = 17,995x + 92,122	0.992	0.416	1.388
*p*-OH-Benzoic acid	y = 16,541x + 145,092	0.976	0.302	1.007
Salicylic acid	y = 32,481x + 226,324	0.986	0.002	0.006
Sinapic acid	y = 3165x + 2785	0.999	0.373	1.245
Syringic acid	y = 1590x − 5036	0.995	0.038	0.126
Vanillic acid	y = 826x − 1108	1.000	0.065	0.218

R^2^—coefficient of determination; LOD—limit of detection (mg/L); LOQ—limit of quantification (mg/L).

## Data Availability

Not applicable.
